# Relationship between Corvis ST tonometry parameters and mean blur rate in the optic nerve head tissue area in healthy eyes

**DOI:** 10.1371/journal.pone.0351637

**Published:** 2026-06-23

**Authors:** Yuta Nakaniida, Fumiko Higashikawa, Kana Tokumo, Shunsuke Nakakura, Ryo Asaoka, Yoshiaki Kiuchi, Hirokazu Sakaguchi

**Affiliations:** 1 Department of Ophthalmology and Visual Science, Hiroshima University, Hiroshima, Japan; 2 Department of Probiotic Science for Preventive Medicine, Graduate School of Biomedical and Health Sciences, Hiroshima University, Hiroshima, Japan; 3 Department of Ophthalmology, Saneikai Tsukazaki Hospital, Himeji, Japan; 4 Department of Ophthalmology, Seirei Hamamatsu General Hospital, Hamamatsu, Shizuoka, Japan; Tsukazaki Hospital, JAPAN

## Abstract

This study investigated the relationship between Corvis ST tonometry parameters and mean blur rate in the optic nerve head tissue area (MBR-T) in healthy eyes. Corneal biomechanical properties and MBR-T were measured in 100 eyes from 56 healthy participants in Japan using Corvis ST tonometry and laser speckle flowgraphy, respectively. Axial length, central corneal thickness, and intraocular pressure were also recorded. Linear mixed-effects models were applied to evaluate the associations between corneal biomechanical properties and MBR-T. Model selection was performed using the second-order bias-corrected Akaike Information Criterion (AICc). The optimal predictive model for MBR-T was as follows: MBR-T = 21.1607 (intercept) – 1.6317 × Sex (Male) – 4.8626 × A2 deformation amplitude – 5.7932 × HC deformation amplitude (AICc = 409.9). In healthy eyes, greater corneal deformability was associated with lower MBR-T.

## Introduction

The development of the Corneal Visualization Scheimpflug Technology (Corvis ST tonometry [CST]; Oculus, Wetzlar, Germany) has enabled direct visualization of corneal movement in response to an air-puff stimulus, facilitating quantitative assessment of corneal biomechanical properties [1]. Previous studies have reported increased corneal deformation in patients with primary open-angle glaucoma (POAG) [2].

Laser speckle flowgraphy (LSFG; Softcare, Fukuoka, Japan) has facilitated the quantitative evaluation of ocular blood flow [3]. LSFG calculates the mean blur rate (MBR), an index reflecting blood flow velocity, by analyzing temporal velocity changes in the speckle pattern. This technique is used to assess blood flow in the optic nerve head (ONH). It has been reported that MBR in the ONH tissue area (MBR-T) is significantly reduced in patients with POAG and pre-perimetric glaucoma compared with that in healthy eyes [4,5].

Greater corneal deformability has been associated with more pronounced posterior bowing of the lamina cribrosa (LC). The LC is a porous connective tissue structure within the ONH that provides structural support for retinal ganglion cell axons. In addition, lower corneal hysteresis has been linked to increased posterior curvature of the LC [6]. Previous studies have suggested that corneal biomechanical properties may be related to ONH structural characteristics, including LC morphology. However, LC morphology was not directly assessed in this study. Therefore, any potential relationship among corneal deformability, LC biomechanics, and MBR-T should be considered hypothetical.

The purpose of this study was to examine the relationship between CST parameters and MBR-T in healthy eyes.

## Materials and methods

The study protocol was reviewed and approved by the Ethical Committee for Epidemiology of Hiroshima University (permission No. E-2587). All participants provided written informed consent allowing their clinical data to be stored in the hospital database and used for research purposes. The study adhered to the tenets of the Declaration of Helsinki.

### Participants

For this retrospective analysis, the authors accessed the study database for research purposes on January 4, 2022. The dataset was analyzed in a de-identified form, and the authors did not have access to information that could directly identify individual participants.

Baseline data from participants enrolled in a study conducted at Hiroshima University between September 10, 2021 and December 25, 2021 were analyzed. A total of 100 eyes from 56 healthy individuals in Japan were included. Eligible participants were those aged ≥20 years who had a best-corrected visual acuity of 20/20 or better, no ocular diseases other than refractive error, systolic blood pressure (SBP) <160 mmHg, and diastolic blood pressure (DBP) <100 mmHg. Exclusion criteria included a history of smoking, refractive surgery, or intraocular surgery.

Mean arterial blood pressure (MAP) was calculated from seated SBP and DBP using the following formula.


$$\begin{array}{c} MAP\;=\;DBP\;+\;\text{1}/\text{3}\;(SBP\;-\;DBP). \end{array}$$

### CST measurement

The operating principle of CST has been described previously [1]. Briefly, the device employs a high-speed Scheimpflug camera to capture a sequence of corneal images during its dynamic response to an air-puff stimulus. It acquires images at 4,330 frames per second, and the recorded data are processed to derive parameters such as central corneal thickness (CCT), deformation amplitude, and length of the deformed corneal region.

The CST parameters were defined as follows: A1 time was defined as the interval from air-puff initiation to the first applanation, corresponding to the initial corneal flattening during inward deformation. In noncontact tonometry, intraocular pressure (IOP) is estimated by converting this first-applanation timing (A1 time) into an IOP value [7–10].

A2 time was defined as the timing of the second applanation, which occurs during the outward rebound of the cornea toward its baseline configuration after inward displacement. Following the first applanation, the cornea continues to move posteriorly before entering the recovery phase.

A1/2 velocity represented the velocity of the corneal apex at the first and second applanations, respectively. A1/2 length referred to the length of the flattened corneal area at A1 and A2 time points, respectively. A1/A2 deformation amplitude indicated the magnitude of deformation at A1 and A2 time points, respectively.

The maximum corneal depression time was defined as the time point at which highest concavity (HC) was achieved. HC length referred to the length of the flattened area at HC time, and HC deformation amplitude represented the deformation magnitude at HC time. Peak distance was defined as the distance between the two peripheral deformation peaks observed at HC time. Radius represented the radius of curvature of the central cornea at HC time. Whole eye movement was defined as the maximum posterior displacement of the entire eye induced by the air-puff stimulus, and whole eye movement time referred to the time required to reach this displacement.

CST examinations (software v1.6r2031) were performed three times per eye on the same day, with a minimum 1-min interval between recordings. The mean value of the three measurements was used for analysis. Only scans that met the device’s on-screen quality criteria (quality index: “OK”) were included.

### LSFG measurements

MBR-T was measured using LSFG (Softcare, Fukuoka, Japan). Blood pressure and heart rate were recorded after participants rested in a seated position for 5 min in a quiet, dark room. The principles of LSFG have been described previously [3]. The LSFG system consists of a diode laser (wavelength: 830 nm) and a fundus camera equipped with a charge-coupled device camera (resolution: 750 × 360 pixels). The accompanying analysis software (LSFG Analyzer, version 3.2.3.0; Softcare, Fukuoka, Japan) automatically identifies the beginning and end of the cardiac cycle from images acquired during a 4-s recording period.

The laser light scattered by the movement of red blood cells produces an interference pattern known as speckle pattern. The rate of change in this pattern is used to calculate MBR, a relative index of blood flow velocity.

In this study, analysis focused on blood flow in the ONH tissue area. The region of interest was defined based on fundus photographs. LSFG Analyzer software automatically segmented the ONH into large vessel and tissue regions and calculated MBR for each region. Although the ONH was further divided into four sectors (superior, inferior, nasal, and temporal), these sectors were combined and analyzed as entire ONH region. MBR-T was calculated by excluding the large vascular regions and summing the blood flow signals from the surface capillaries to the cribriform plate within the nonretinal vascular ONH tissue area.

The average value of three LSFG measurements was used for analysis. The “follow-up scan” function of LSFG Analyzer was employed to ensure consistent measurement location and size across repeated scans for each participant.

### Other measurements

Axial length (AL) was measured using an IOL Master 700 (Carl Zeiss Meditec, Dublin, CA, USA). IOP was measured using a Goldmann applanation tonometer.

### Statistical analysis

Both eyes from each participant were included in the analysis. Therefore, linear mixed-effects models (LMMs) [11,12] were used, with subject ID treated as a random effect.

MBR-T was designated as the objective variable. Six basic (age, sex, AL, IOP, CCT, and MAP) and 15 CST parameters (A1/2 time, A1/2 velocity, A1/2 length, A1/2 deformation amplitude, HC time, HC length, HC deformation amplitude, peak distance, radius, whole eye movement, and whole eye movement time) were considered explanatory variables. The optimal model (Model^Basic^ and Model^Basic_CST^) for MBR-T was selected from all possible combinations of parameters using the second-order bias-corrected Akaike Information Criterion (AICc). AICc is a small-sample correction of the Akaike Information Criterion (AIC) and provides more reliable estimates when sample sizes are limited [13].

Before the AICc-based model selection, associations between MBR-T and 6 basic and 15 CST parameters were first evaluated using single-parameter LMMs, with subject ID included as a random effect. The model was specified as follows:


$$\begin{array}{c} MBR-T\;=\;{\text{Parameter}}_{\text{i}}\;+\;(\text{1}\;|\;Subject\;ID). \end{array}$$

Where Parameterᵢ represents 6 basic and 15 CST parameters examined separately.

First, the optimal basic model (Model^Basic^) for MBR-T was selected from all possible combinations of the 6 basic parameters (2^6^ = 64 candidate models) using AICc.

To account for potential confounding, adjusted LMMs were constructed using clinically relevant covariates as fixed effects:

MBR−T = Age + Sex + AL + IOP + CCT + MAP + (1 | Subject ID).

Second, a model incorporating both basic and CST parameters (Model^Basic_CST^) was selected from all possible combinations of 21 variables (6 basic + 15 CST; 2^21^ = 2,097,152 candidate models) based on AICc. An adjusted full model was also constructed as follows:


$$\begin{array}{cc} MBR-T\;= & \;Age\;+\;Sex\;+\;AL\;+\;IOP\;+\;CCT\;+\;MAP\;+\;A\text{1}\;time\;+\;A\text{1}\;velocity\;\\ & +\;A\text{1}\;length\;+\;A\text{1}deformation\;amplitude\;+\;A\text{2}\;time\;+\;A\text{2}\;velocity\;+\;A\text{2}\;length\;\\ & +\;A\text{2}deformation\;amplitude\;+\;HC\;time\;+\;HC\;length\;+\;HC\;deformation\;amplitude\;\\ & +\;Peak\;distance\;+\;Radius\;+\;Whole\;eye\;movement\;\\ & +\;Whole\;eye\;movement\;time\;+\;(\text{1}\;|\;Subject\;ID). \end{array}$$

In multivariate regression analysis, inclusion of excessive predictors reduces degrees of freedom and may impair model performance. Therefore, model selection procedures were used to eliminate redundant variables and improve model fit [14,15]. A lower AICc value indicates a better-fitting model.

The relative likelihood of each candidate model was calculated as follows: exp((AIC_min_ − AIC_i_)/2), where AIC_min_ represents the smallest AICc value among the candidate models. This value reflects the probability that a given model minimizes information loss and is therefore the best model [16]. Relative probabilities were computed for all candidate models.

All statistical analyses were performed using R (version 4.5.2; R Foundation for Statistical Computing, Vienna, Austria).

## Results

The characteristics of the study participants are summarized in Table 1. Values are presented as means  ±  standard deviations (SDs). The mean age was 51.67  ± 10.27 years. Overall, 11 participants were male, and 45 were female. The SBP was 106.76  ±  16.98 mmHg, and DBP was 63.35  ±  12.13 mmHg. The mean arterial blood pressure was 77.82  ±  13.41 mmHg, AL was 24.54  ±  1.44 mm, IOP was 11.89  ±  2.49 mmHg, CCT was 553.40  ±  35.17 µm, and MBR-T was 12.53  ±  2.78 arbitrary units.

**Table 1:** Characteristics of the participants.

Parameter	Mean (SD) [Range]
Number of participants	56
Number of eyes	100
Age (years)	51.67 (10.27) [20.00–71.00]
Sex (male/female)	11/45
Axial length (mm)	24.54 (1.44) [21.49–28.21]
Intraocular pressure (mmHg)	11.89 (2.49) [7.00–20.00]
Central corneal thickness (µm)	553.40 (35.17) [469.67–675.00]
Systolic blood pressure (mmHg)	106.76 (16.98) [76.00–159.50]
Diastolic blood pressure (mmHg)	63.35 (12.13) [32.50–95.50]
Mean arterial blood pressure (mmHg)	77.82 (13.41) [47.00–115.00]
MBR-T (arbitrary unit)	12.53 (2.78) [8.50–19.10]

Abbreviations: MBR-T, mean blur rate in the optic nerve head tissue area; SD, standard deviation.

Summary statistics for the 15 CST parameters are provided in Table 2.

**Table 2:** Summary statistics of the 15 CST parameters.

Parameter	Mean (SD) [Range]
A1 time (ms)	7.29 (0.26) [6.71–8.18]
A1 velocity (m/s)	0.15 (0.01) [0.11–0.19]
A1 length (mm)	2.26 (0.13) [2.01–2.62]
A1 deformation amplitude (mm)	0.13 (0.01) [0.11–0.15]
A2 time (ms)	22.16 (0.34) [21.44–23.25]
A2 velocity (m/s)	−0.27 (0.03) [−0.35 – −0.19]
A2 length (mm)	2.84 (0.45) [2.09–3.87]
A2 deformation amplitude (mm)	0.42 (0.06) [0.29–0.55]
HC time (ms)	17.26 (0.36) [16.40–18.04]
HC length (mm)	6.43 (0.39) [5.62–7.81]
HC deformation amplitude (mm)	1.10 (0.09) [0.89–1.35]
Peak distance (mm)	4.98 (0.25) [4.45–5.73]
Radius (mm)	6.91 (0.67) [5.30–9.12]
Whole eye movement (mm)	0.31 (0.06) [0.19–0.44]
Whole eye movement time (ms)	22.13 (0.61) [21.15–24.03]

Abbreviations: A1, applanation first; A2, applanation second; HC, highest concavity; SD, standard deviation; CST, Corvis ST tonometry.

The results of the single-parameter LMMs assessing associations of MBR-T with 6 basic and 15 CST parameters are shown in Table 3. Significant associations were observed for IOP, CCT, A2 length, and HC deformation amplitude. IOP showed a positive association with MBR-T (coefficient = 0.2646, p = 0.0060), whereas CCT, A2 length, and HC deformation amplitude showed negative associations with MBR-T (CCT: coefficient = −0.0195, p = 0.0397; A2 length: coefficient = −0.5978, p = 0.0477; HC deformation amplitude: coefficient = −7.1003, p = 0.0183).

**Table 3:** Single-parameter LMMs for MBR-T.

Parameters	Coefficient	Standard error	*p*-value	AICc
Age	−0.0254	0.0349	0.4688	424.4
Sex	−1.6284	0.8767	0.0686	420.2
AL	−0.0575	0.2354	0.8079	424.9
**IOP**	**0.2646**	**0.0940**	**0.0060**	**420.6**
**CCT**	**−0.0195**	**0.0093**	**0.0397**	**420.8**
MAP	−0.0386	0.0268	0.156	423.0
A1 time	2.0166	1.0411	0.0556	421.5
A1 velocity	2.3376	16.9604	0.8907	425.0
A1 length	0.9826	1.6063	0.5425	424.7
A1 deformation amplitude	0.2346	23.8171	0.9922	425.0
A2 time	−1.1208	0.6642	0.0952	422.3
A2 velocity	16.0318	8.6326	0.0666	421.6
**A2 length**	**−0.5978**	**0.2948**	**0.0477**	**420.9**
A2 deformation amplitude	−6.3630	3.9155	0.1076	422.4
HC time	0.1142	0.4897	0.8163	424.9
HC length	−0.8019	0.5908	0.1781	423.2
**HC deformation amplitude**	**−7.1003**	**2.9601**	**0.0183**	**419.9**
Peak distance	−1.9064	1.0468	0.0717	421.8
Radius	−0.0780	0.3163	0.8059	424.9
Whole eye movement	−2.7940	4.1097	0.4983	424.5
Whole eye movement time	0.3224	0.3215	0.3191	423.9

Bold characters represent *p*  <  0.05.

CST, Corvis ST tonometry; AL, axial length; HC, highest concavity; IOP, intraocular pressure; CCT, central corneal thickness; MBR-T, mean blur rate in the optic nerve head tissue area; AICc, second-order bias-corrected Akaike Information Criterion.

Based on model selection, the optimum model for Model^Basic^ was as follows:


$$\begin{array}{c} MBR-T\;=\;\;\text{9}.\text{7}\text{8}\text{9}\text{8}\;(intercept)\;-\;\text{1}.\text{2}\text{1}\text{9}\text{4}\;\times \;Sex\;(Male)\;+\;\text{0}.\text{2}\text{4}\text{0}\text{1}\;\times \;IOP\;(AICc\;=\;\text{4}\text{1}\text{9}.\text{6}). \end{array}$$

The optimum model for Model^Basic_CST^ was as follows:


$$\begin{array}{cc} MBR-T\;= & \;\text{2}\text{1}.\text{1}\text{6}\text{0}\text{7}\;(intercept)-\;\text{1}.\text{6}\text{3}\text{1}\text{7}\;\\ & \times \;Sex\;(Male)-\;\text{4}.\text{8}\text{6}\text{2}\text{6}\;\times \;A\text{2}\;deformation\;amplitude\;-\;\text{5}.\text{7}\text{9}\text{3}\text{2}\;\\ & \times \;HC\;deformation\;amplitude \end{array}$$

$$\begin{array}{c} (AICc\;=\;\text{4}\text{0}\text{9}.\text{9}). \end{array}$$

The AICc values were 419.6 for Model^Basic^ and 409.9 for Model^Basic_CST^. The difference in AICc was calculated as follows: ΔAICc = 419.6 − 409.9 = 9.7. Model^Basic_CST^ had a lower AICc than Model^Basic^ (409.9 vs 419.6; ΔAICc = 9.7), suggesting strong support for including CST parameters into the model. In this two-model comparison, the Akaike weight for Model^Basic_CST^ was 0.992. The relative probability that Model^Basic_CST^ minimized information loss within this two-model comparison was calculated using the Akaike weight formula:


$$\begin{array}{c} w\;=\;\frac{\text{1}}{\text{1}+\text{exp}(-\Delta \text{AICc}/\text{2})} \end{array}$$

Substituting ΔAICc = 9.7 gives:


$$\begin{array}{c} w\;=\;\frac{\text{1}}{\text{1}+\text{exp}(-\text{9}.\text{7}/\text{2})}\;=\frac{\text{1}}{\text{1}+\text{0}.\text{0}\text{0}\text{7}\text{8}}\;=\;\text{0}.\text{9}\text{9}\text{2} \end{array}$$

Therefore, the relative probability that Model^Basic_CST^ minimized information loss was 99.2%.

## Discussion

In this study, single-parameter LMMs showed that MBR-T was significantly associated with IOP, CCT, A2 length, and HC deformation amplitude. In the AICc-based model selection, sex (male) and IOP were selected in Model^Basic^, whereas sex (male), A2 deformation amplitude, and HC deformation amplitude were selected in Model^Basic_CST^. These findings suggest that corneal biomechanical properties, particularly parameters related to greater corneal deformation, may be associated with lower MBR-T in healthy eyes.

In the single-parameter LMMs, lower IOP, greater CCT, longer A2 length, and greater HC deformation amplitude were associated with lower MBR-T. The positive association between IOP and MBR-T should be interpreted cautiously. Although IOP showed a positive association with MBR-T in the single-parameter LMMs and was retained in Model^Basic,^ it was not retained in Model^Basic_CST^. Because all participants were healthy and the IOP range was within a relatively physiological range, this finding may reflect interindividual variation or confounding by systemic circulatory factors rather than a direct causal relationship. The negative association between CCT and MBR-T should be interpreted cautiously. This association was observed in the single-parameter LMMs, but CCT was not retained in Model^Basic^ and Model^Basic_CST^. Therefore, we do not interpret CCT as having a direct independent effect on ONH tissue blood flow. Rather, the association may reflect confounding or shared variance with IOP and corneal biomechanical properties, because CCT can influence tonometric measurements and is related to the biomechanical response of the cornea. A2 length and HC deformation amplitude showed significant associations with MBR-T among 15 CST parameters. In Model^Basic_CST^, A2 length was not retained, whereas A2 deformation amplitude and HC deformation amplitude were selected. Although A2 length and A2 deformation amplitude represent different aspects of the second applanation phase, both longer A2 length and greater A2 deformation amplitude indicate greater corneal deformation. These results suggest that greater corneal deformation is associated with lower MBR-T.

In Model^Basic^, sex (male) and lower IOP were associated with reduced MBR-T. Male participants tended to have lower MBR-T than females participants, and IOP showed a positive association with MBR-T. Sex was retained in the selected models; however, because the number of male participants was limited, this finding should be interpreted as an adjustment factor or exploratory observation rather than evidence of a definitive sex difference. A previous study reported sex-related differences in MBR-T [17]. Therefore, sex was included as a clinically relevant covariate in the present analysis. However, because only 11 of the 56 participants were male, the present study was not sufficiently powered to draw definitive conclusions regarding sex differences in MBR-T.

In Model^Basic_CST^, sex (male), greater A2 deformation amplitude and HC deformation amplitude were associated with reduced MBR-T. Greater A2 deformation amplitude and HC deformation amplitude indicate that MBR-T is lower in eyes that exhibit greater corneal indentation. Collectively, these findings suggest that the depth of corneal deformation was related to reduced ONH blood flow.

Moreover, Model^Basic_CST^ had a lower AICc than Model^Basic^, with an Akaike weight of 0.992 in this two-model comparison, suggesting that inclusion of CST parameters improved model fit for MBR-T. The Model^Basic_CST^ further suggests that MBR-T is reduced in eyes with corneas that deform more deeply.

Although many previous reports have focused on POAG eyes, it has been shown that eyes with more easily deformable corneas tend to exhibit faster progression of visual field loss [18]. Untreated POAG eyes have also been reported to have more deformable corneas than healthy eyes [2]. Furthermore, MBR-T is significantly lower in POAG eyes than in healthy eyes [4]. Even in pre-perimetric glaucoma, in which patients show no subjective symptoms or visual field defects but exhibit glaucomatous structural changes on OCT, MBR-T has been reported to be reduced relative to healthy eyes [5]. Taken together, these findings suggest that POAG eyes are characterized by both increased corneal deformability and reduced MBR-T. Therefore, the present observation that greater corneal deformability is associated with lower MBR-T even in healthy eyes is consistent with existing literature.

Lower corneal stiffness has also been associated with increased posterior LC curvature [19]. The LC is a porous connective tissue scaffold within the ONH that provides structural support for retinal ganglion cell axons. In POAG eyes, posterior bowing and thinning of the LC are believed to contribute to glaucomatous optic neuropathy and visual field loss [20]. It has been reported that lower corneal hysteresis and higher IOP are associated with greater posterior bowing of the LC [6]. Although previous studies have reported associations between corneal biomechanical properties and LC morphology, this study did not directly evaluate LC depth, curvature, or thickness. Therefore, the possible relationship among greater corneal deformability, LC biomechanics, and lower MBR-T should be regarded as a hypothesis rather than a direct finding of this study.

This study has several limitations. First, only healthy eyes were included; therefore, the findings cannot be directly extrapolated to patients with glaucoma. Second, the number of male participants was limited, and the sex distribution was imbalanced. Therefore, the present study was not designed or powered to evaluate sex differences in MBR-T, and sex-related estimates should be interpreted cautiously. Third, the cross-sectional design precludes determination of causal relationships. Fourth, although IOP and MBR-T are influenced by diurnal variation and systemic circulatory factors, such as blood pressure, pulse rate, and ocular perfusion pressure, these variables may not have been adequately controlled. Fifth, corneal hysteresis was not assessed in this study. Corneal hysteresis is an important parameter reflecting corneal viscoelasticity and has been associated with glaucoma-related structural changes. Future studies incorporating both CST parameters and Ocular Response Analyzer-derived corneal hysteresis are needed to determine whether corneal hysteresis is associated with MBR-T. Sixth, the relationship between CCT and MBR-T could not be fully separated from CST parameters in this study. Seventh, LC morphology was not directly assessed in this study. Future studies using OCT-based measurements of LC depth, curvature, and thickness are needed to clarify whether LC biomechanics mediate the relationship between corneal deformability and ONH tissue blood flow. Eighth, detailed information on systemic diseases and systemic medication use was not available for all participants. This is a limitation because MBR-T may be influenced by systemic circulatory status and vasoactive medications, including antihypertensive, antidiabetic, lipid-lowering, antiplatelet, anticoagulant, and other vasoactive agents. Ninth, we evaluated overall MBR-T and did not assess regional peripapillary choroidal perfusion patterns. Recent LSFG-based evidence suggests that the spatial distribution of choroidal watershed zones or peripapillary choroidal hypoperfusion zones may influence sectoral ONH tissue blood flow [21]. Therefore, regional choroidal perfusion patterns may represent an unmeasured factor affecting overall MBR-T in the present study. Future studies incorporating sectoral MBR-T analysis and peripapillary choroidal perfusion mapping are needed.

In conclusion, in healthy eyes, CST parameters were associated with MBR-T. CST parameters indicative of greater corneal deformation were associated with lower MBR-T.

## Supporting information

S1 Data Anonymized dataset used in this study, excluding the Age variable to protect participant privacy. (XLSX)
