# Supplementary material for: Relationship between Corvis ST tonometry parameters and mean blur rate in the optic nerve head tissue area in healthy eyes
Source: PLoS One. 2026 Jun 23;21(6):e0351637. doi: 10.1371/journal.pone.0351637 (PMC13289879; doi:10.1371/journal.pone.0351637)
Supplement: Attachment — Submitted filename: 02_Response to Reviewers.docx [file pone.0351637.s003.docx]

May 13, 2026

Daisuke Nagasato

Academic Editor

*PLOS One*

**Response to Reviewers**

Manuscript title: **Relationship between Corvis ST tonometry parameters and mean blur rate in the optic nerve head tissue area in healthy eyes**

Submission ID: **PONE-D-26-08321**

Dear Academic Editor and Reviewers,

We sincerely thank the Academic Editor and the Reviewers for their careful evaluation of our manuscript and for their constructive comments. In response to these valuable suggestions, we have revised the manuscript accordingly, and we believe that these revisions have substantially improved its clarity, transparency, and interpretability. Our “Response to Reviewers” is provided below. Reviewer comments are shown in bold, followed by our responses.

We also reran the model-selection analysis after adding MAP and correcting the coefficient extraction; consequently, the selected Model^Basic_CST^ was updated to include sex, A2 deformation amplitude, and HC deformation amplitude. The Abstract, Results, and Discussion were revised accordingly.

In accordance with the comments from PLOS ONE, we removed the Age column from the publicly shared dataset to protect participant privacy.

We also corrected errors in the signs of the coefficients for IOP and A1 length in Table 3. Specifically, in the single-parameter LMMs, the coefficients for both IOP and A1 length were positive, not negative. The corresponding descriptions in the Results and Discussion sections have been revised accordingly to ensure consistency with the corrected Table 3 and to avoid misinterpretation.

**Reviewer #1 Comment**

**This manuscript investigates the correlation between parameters obtained from Corvis ST (CST) and MBR-T measured by LSFG. However, there are several concerns regarding the study design and the interpretation of the results.**

Response: We sincerely thank the reviewer for the careful and constructive evaluation of our manuscript. We appreciate the reviewer’s important comments regarding the study design and interpretation of the results. These comments were very helpful for improving the clarity and scientific rigor of our manuscript. We have carefully addressed each point and revised the manuscript accordingly.

**Comment 1) The manuscript mentions sex differences, but the sample size appears insufficient for this analysis. Please either increase the number of male subjects or provide justification that the current sample size is adequate for a valid comparison.**

Response: Thank you for this important comment. We agree that the number of male participants was limited and that the present cohort was not sufficiently balanced to allow a definitive analysis of sex differences. Our intention was not to perform a sex-specific comparison, but to include sex as a clinically relevant covariate, because previous LSFG studies have reported sex-related differences in ocular blood flow.

To avoid overinterpretation, we have revised the Results, Discussion, and Conclusion to clarify that sex was treated as an adjustment covariate and that sex-related findings should be interpreted as exploratory. We have also added this issue to the limitations, stating that the study was not designed or powered to evaluate sex differences because of the limited number of male participants.

**Comment 2) Why was corneal hysteresis excluded from this study? Compared with previous studies, it would be more interesting to examine whether corneal hysteresis correlates with MBR-T, rather than focusing only on individual parameters obtained from CST.**

Response: Thank you for this important comment. We agree with the reviewer that corneal hysteresis is an important biomechanical parameter and that its relationship with MBR-T would be of considerable interest. In the present retrospective dataset, however, Ocular Response Analyzer measurements were not available, and therefore corneal hysteresis could not be analyzed.

We have added the absence of corneal hysteresis measurements as a limitation and stated that future studies incorporating both CST parameters and Ocular Response Analyzer-derived corneal hysteresis are needed.

**Comment 3) How do the authors interpret the finding that greater central corneal thickness is associated with a decrease in MBR-T?**

Response: Thank you for raising this point. We agree that the association between greater CCT and lower MBR-T requires careful interpretation. In the present analysis, CCT showed a negative association with MBR-T in the single-parameter LMM; however, CCT was not retained in the final AICc-selected model that included CST parameters.

Therefore, we do not interpret CCT as having a direct independent effect on ONH tissue blood flow. We have revised the Discussion to state that this finding may reflect confounding or shared variance with IOP and corneal biomechanical properties, and that the relationship between CCT and MBR-T should be interpreted cautiously.

**Comment 4) How was blood pressure handled in this study? Should it not be considered as a potential factor associated with MBR-T?**

Response: Thank you for this important comment. We agree that blood pressure may influence MBR-T. In the revised analysis, we added mean arterial blood pressure, calculated from seated systolic blood pressure and diastolic blood pressure, as one of the six basic parameters. MAP was included in the single-parameter LMMs and in the AICc-based model selection for both Model^Basic^ and Model^Basic_CST^. In the single-parameter LMMs, MAP was not significantly associated with MBR-T (coefficient = −0.0386, p = 0.156). MAP was not retained in the final selected Model^Basic^ and Model^Basic_CST^. We revised the Methods, Table 1, Table 3, Results, and Discussion accordingly. We also added insufficient control of systemic circulatory factors, including blood pressure and ocular perfusion pressure, as a limitation.

**Reviewer #2 Comment**

**This study investigated the relationship between Corvis ST tonometry parameters and MBR-T measured by LSFG in healthy eyes. The topic is very interesting, and the combination of corneal biomechanical assessment and optic nerve head blood flow analysis is valuable. Overall, the manuscript is well organized. However, several minor points should be addressed to improve the clarity and completeness of the manuscript before publication.**

Response: We sincerely thank the reviewer for the careful evaluation of our manuscript and for the encouraging comments. We are grateful for the reviewer’s positive assessment that the topic is interesting, that the combination of corneal biomechanical assessment and optic nerve head blood flow analysis is valuable, and that the manuscript is well organized. We also appreciate the constructive suggestions for improving the clarity and completeness of the manuscript. We have carefully addressed each point and revised the manuscript accordingly.

**Comment 1) Please correct the inconsistency in the number of participants. The submission abstract states that the study included 100 eyes from 50 healthy participants, whereas the manuscript text and Table 1 state 100 eyes from 56 participants. This discrepancy should be corrected throughout the manuscript and submission documents.**

Response: Thank you for pointing out this inconsistency. The correct number of participants is 56. We have corrected the submission abstract, cover letter, and manuscript text so that the study population is consistently described as 100 eyes from 56 healthy participants throughout the submission.

**Comment 2) The discussion about lamina cribrosa biomechanics is interesting and relevant. However, lamina cribrosa morphology was not directly evaluated in this study. Therefore, the authors should describe this interpretation more cautiously. The possible relationship among corneal deformability, lamina cribrosa biomechanics, and lower MBR-T should be presented as a hypothesis rather than a direct finding of this study. This point should also be added to the limitations. Future studies using OCT-based assessment of lamina cribrosa morphology, such as lamina cribrosa depth, curvature, or thickness, may help clarify the mechanism.**

Response: We thank the reviewer for this helpful suggestion. We agree that lamina cribrosa morphology was not directly evaluated in the present study and that the relationship among corneal deformability, lamina cribrosa biomechanics, and lower MBR-T should not be presented as a direct finding.

We have revised the Introduction and Discussion to describe this relationship more cautiously and to present it as a hypothesis. We have also added this point to the limitations, stating that future studies using OCT-based assessment of lamina cribrosa depth, curvature, and thickness are needed to clarify the underlying mechanism.

**Comment 3) The manuscript describes the participants as healthy, but it does not clearly state whether systemic diseases or medication use were assessed. Because MBR-T may be influenced by systemic circulatory status and vasoactive medications, the authors should clarify this point. If participants were confirmed not to be taking systemic medications, such as antihypertensive agents, antidiabetic drugs, lipid-lowering drugs, antiplatelet/anticoagulant agents, or other vasoactive medications, this should be clearly stated in the inclusion or exclusion criteria. If information on medication use was not available, this should be acknowledged as a limitation.**

Response: Thank you for this important comment. We agree that systemic diseases and systemic medication use may influence MBR-T. In the present retrospective dataset, detailed information regarding systemic diseases and medication use was not available for all participants. Therefore, we have added this issue to the limitations, stating that systemic circulatory status and vasoactive medications, including antihypertensive agents, antidiabetic drugs, lipid-lowering drugs, antiplatelet or anticoagulant agents, and other vasoactive medications, may have influenced MBR-T.

**Comment 4) The present study evaluated MBR-T, but regional peripapillary choroidal perfusion patterns were not assessed. Recent LSFG-based evidence suggests that the spatial distribution of choroidal watershed zones or peripapillary choroidal hypoperfusion zones may influence sectoral ONH tissue blood flow. Therefore, these regional perfusion patterns may be an unmeasured factor affecting overall MBR-T. The authors should consider adding this point to the limitations or future directions. Relevant recent work may be cited if appropriate: Hashimoto R, Kardon RH. Laser speckle flowgraph reveals dynamic characteristics and clinical relevance of choroidal watershed and peripapillary hypoperfusion zones. Scientific Reports. 2026. doi: 10.1038/s41598-026-47062-z.**

Response: Thank you for this valuable suggestion. We agree that regional peripapillary choroidal perfusion patterns may influence ONH tissue blood flow and that this issue was not evaluated in the present study.

We have added this point to the limitations and future directions. Specifically, we now state that the present study evaluated overall MBR-T and did not assess the spatial distribution of choroidal watershed zones or peripapillary choroidal hypoperfusion zones. We have also cited the recent LSFG-based study by Hashimoto and Kardon, as suggested by the reviewer, and noted that future studies incorporating sectoral MBR-T analysis and peripapillary choroidal perfusion mapping are needed.

We hope that the manuscript is now suitable for publication in *PLOS ONE*.

Sincerely,

Yuta Nakaniida

Department of Ophthalmology and Visual Science, Hiroshima University, 1-2-3 Kasumi, Minami-ku, Hiroshima, 734-8551, Japan

Tel:+81-822575247

Fax:+81-822575249

E-mail: ynakaniida@outlook.jp

ORCID: 0000-0002-2778-1017
