# Supplementary material for: Relationship between Corvis ST tonometry parameters and mean blur rate in the optic nerve head tissue area in healthy eyes
Source: PLoS One. 2026 Jun 23;21(6):e0351637. doi: 10.1371/journal.pone.0351637 (PMC13289879; doi:10.1371/journal.pone.0351637)
Supplement: Peer review history

# Peer review: Decision Letter 0

**DOI:** 10.1371/journal.pone.0351637.r001

**Authors:** Daisuke Nagasato

5 May 2026

PONE-D-26-08321

Relationship between Corvis ST tonometry parameters and mean blur rate in the optic nerve head tissue area in healthy eyes

PLOS One

Dear Dr. Nakaniida,

Thank you for submitting your manuscript to PLOS ONE. After careful consideration, we feel that it has merit but does not fully meet PLOS ONE’s publication criteria as it currently stands. Therefore, we invite you to submit a revised version of the manuscript that addresses the points raised during the review process.

Please submit your revised manuscript by Jun 19 2026 11:59PM. If you will need more time than this to complete your revisions, please reply to this message or contact the journal office at plosone@plos.org>. When you're ready to submit your revision, log on to https://www.editorialmanager.com/pone/ and select the 'Submissions Needing Revision' folder to locate your manuscript file.

Please include the following items when submitting your revised manuscript:

- A letter that responds to each point raised by the academic editor and reviewer(s). You should upload this letter as a separate file labeled 'Response to Reviewers'.
- A marked-up copy of your manuscript that highlights changes made to the original version. You should upload this as a separate file labeled 'Revised Manuscript with Track Changes'.
- An unmarked version of your revised paper without tracked changes. You should upload this as a separate file labeled 'Manuscript'.

If you would like to make changes to your financial disclosure, please include your updated statement in your cover letter. Guidelines for resubmitting your figure files are available below the reviewer comments at the end of this letter.

If applicable, we recommend that you deposit your laboratory protocols in protocols.io to enhance the reproducibility of your results. Protocols.io assigns your protocol its own identifier (DOI) so that it can be cited independently in the future. For instructions see: https://journals.plos.org/plosone/s/submission-guidelines#loc-laboratory-protocols. Additionally, PLOS ONE offers an option for publishing peer-reviewed Lab Protocol articles, which describe protocols hosted on protocols.io. Read more information on sharing protocols at https://plos.org/protocols?utm_medium=editorial-email&utm_source=authorletters&utm_campaign=protocols.

As the corresponding author, your ORCID iD is verified in the submission system and will appear in the published article. PLOS supports the use of ORCID, and we encourage all coauthors to register for an ORCID iD and use it as well. Please encourage your coauthors to verify their ORCID iD within the submission system before final acceptance, as unverified ORCID iDs will not appear in the published article. *Only*  the individual author can complete the verification step; PLOS staff *cannot*  verify ORCID iDs on behalf of authors.

We look forward to receiving your revised manuscript.

Kind regards,

Daisuke Nagasato

Academic Editor

PLOS One

Journal Requirements:

When submitting your revision, we need you to address these additional requirements.

1. Please ensure that your manuscript meets PLOS ONE's style requirements, including those for file naming. The PLOS ONE style templates can be found at

https://journals.plos.org/plosone/s/file?id=wjVg/PLOSOne_formatting_sample_main_body.pdf and

https://journals.plos.org/plosone/s/file?id=ba62/PLOSOne_formatting_sample_title_authors_affiliations.pdf

2. We note that there is identifying data in the Supporting Information file <03_DataSet_100Healthy_CST_MBR-T>. Due to the inclusion of these potentially identifying data, we have removed this file from your file inventory. Prior to sharing human research participant data, authors should consult with an ethics committee to ensure data are shared in accordance with participant consent and all applicable local laws.

Data sharing should never compromise participant privacy. It is therefore not appropriate to publicly share personally identifiable data on human research participants. The following are examples of data that should not be shared:

-Name, initials, physical address

-Ages more specific than whole numbers

-Internet protocol (IP) address

-Specific dates (birth dates, death dates, examination dates, etc.)

-Contact information such as phone number or email address

-Location data

-ID numbers that seem specific (long numbers, include initials, titled “Hospital ID”) rather than random (small numbers in numerical order)

Data that are not directly identifying may also be inappropriate to share, as in combination they can become identifying. For example, data collected from a small group of participants, vulnerable populations, or private groups should not be shared if they involve indirect identifiers (such as sex, ethnicity, location, etc.) that may risk the identification of study participants.

Additional guidance on preparing raw data for publication can be found in our Data Policy (https://journals.plos.org/plosone/s/data-availability#loc-human-research-participant-data-and-other-sensitive-data) and in the following article: http://www.bmj.com/content/340/bmj.c181.long.

Please remove or anonymize all personal information (Age), ensure that the data shared are in accordance with participant consent, and re-upload a fully anonymized data set. Please note that spreadsheet columns with personal information must be removed and not hidden as all hidden columns will appear in the published file.

3. If the reviewer comments include a recommendation to cite specific previously published works, please review and evaluate these publications to determine whether they are relevant and should be cited. There is no requirement to cite these works unless the editor has indicated otherwise.

[Note: HTML markup is below. Please do not edit.]

Reviewers' comments:

Reviewer's Responses to Questions

**Comments to the Author**

1. Is the manuscript technically sound, and do the data support the conclusions?

The manuscript must describe a technically sound piece of scientific research with data that supports the conclusions. Experiments must have been conducted rigorously, with appropriate controls, replication, and sample sizes. The conclusions must be drawn appropriately based on the data presented.

Reviewer #1: Partly

Reviewer #2: Yes

**********

2. Has the statistical analysis been performed appropriately and rigorously?

Reviewer #1: Yes

Reviewer #2: Yes

**********

3. Have the authors made all data underlying the findings in their manuscript fully available?

The PLOS Data policy requires authors to make all data underlying the findings described in their manuscript fully available without restriction, with rare exception (please refer to the Data Availability Statement in the manuscript PDF file). The data should be provided as part of the manuscript or its supporting information, or deposited to a public repository. For example, in addition to summary statistics, the data points behind means, medians and variance measures should be available. If there are restrictions on publicly sharing data—e.g. participant privacy or use of data from a third party—those must be specified.

Reviewer #1: Yes

Reviewer #2: Yes

**********

4. Is the manuscript presented in an intelligible fashion and written in standard English?

PLOS ONE does not copyedit accepted manuscripts, so the language in submitted articles must be clear, correct, and unambiguous. Any typographical or grammatical errors should be corrected at revision, so please note any specific errors here.

Reviewer #1: Yes

Reviewer #2: Yes

**********

5. Review Comments to the Author

Please use the space provided to explain your answers to the questions above. You may also include additional comments for the author, including concerns about dual publication, research ethics, or publication ethics. (Please upload your review as an attachment if it exceeds 20,000 characters)

Reviewer #1: This manuscript investigates the correlation between parameters obtained from Corvis ST (CST) and MBR-T measured by LSFG. However, there are several concerns regarding the study design and the interpretation of the results.

• The manuscript mentions sex differences, but the sample size appears insufficient for this analysis. Please either increase the number of male subjects or provide justification that the current sample size is adequate for a valid comparison.

• Why was corneal hysteresis excluded from this study? Compared with previous studies, it would be more interesting to examine whether corneal hysteresis correlates with MBR-T, rather than focusing only on individual parameters obtained from CST.

• How do the authors interpret the finding that greater central corneal thickness is associated with a decrease in MBR-T?

• How was blood pressure handled in this study? Should it not be considered as a potential factor associated with MBR-T?

Reviewer #2: This study investigated the relationship between Corvis ST tonometry parameters and MBR-T measured by LSFG in healthy eyes. The topic is very interesting, and the combination of corneal biomechanical assessment and optic nerve head blood flow analysis is valuable. Overall, the manuscript is well organized. However, several minor points should be addressed to improve the clarity and completeness of the manuscript before publication.

1. Please correct the inconsistency in the number of participants. The submission abstract states that the study included 100 eyes from 50 healthy participants, whereas the manuscript text and Table 1 state 100 eyes from 56 participants. This discrepancy should be corrected throughout the manuscript and submission documents.

2. The discussion about lamina cribrosa biomechanics is interesting and relevant. However, lamina cribrosa morphology was not directly evaluated in this study. Therefore, the authors should describe this interpretation more cautiously. The possible relationship among corneal deformability, lamina cribrosa biomechanics, and lower MBR-T should be presented as a hypothesis rather than a direct finding of this study. This point should also be added to the limitations. Future studies using OCT-based assessment of lamina cribrosa morphology, such as lamina cribrosa depth, curvature, or thickness, may help clarify the mechanism.

3.The manuscript describes the participants as healthy, but it does not clearly state whether systemic diseases or medication use were assessed. Because MBR-T may be influenced by systemic circulatory status and vasoactive medications, the authors should clarify this point. If participants were confirmed not to be taking systemic medications, such as antihypertensive agents, antidiabetic drugs, lipid-lowering drugs, antiplatelet/anticoagulant agents, or other vasoactive medications, this should be clearly stated in the inclusion or exclusion criteria. If information on medication use was not available, this should be acknowledged as a limitation.

4. The present study evaluated MBR-T, but regional peripapillary choroidal perfusion patterns were not assessed. Recent LSFG-based evidence suggests that the spatial distribution of choroidal watershed zones or peripapillary choroidal hypoperfusion zones may influence sectoral ONH tissue blood flow. Therefore, these regional perfusion patterns may be an unmeasured factor affecting overall MBR-T. The authors should consider adding this point to the limitations or future directions. Relevant recent work may be cited if appropriate: Hashimoto R, Kardon RH. Laser speckle flowgraph reveals dynamic characteristics and clinical relevance of choroidal watershed and peripapillary hypoperfusion zones. Scientific Reports. 2026. doi: 10.1038/s41598-026-47062-z.

**********

6. PLOS authors have the option to publish the peer review history of their article (what does this mean?). If published, this will include your full peer review and any attached files.

If you choose “no”, your identity will remain anonymous but your review may still be made public.

**Do you want your identity to be public for this peer review?**  For information about this choice, including consent withdrawal, please see our Privacy Policy.

Reviewer #1: No

Reviewer #2: No

**********

[NOTE: If reviewer comments were submitted as an attachment file, they will be attached to this email and accessible via the submission site. Please log into your account, locate the manuscript record, and check for the action link "View Attachments". If this link does not appear, there are no attachment files.]

To ensure your figures meet our technical requirements, please review our figure guidelines: https://journals.plos.org/plosone/s/figures

You may also use PLOS’s free figure tool, NAAS, to help you prepare publication quality figures: https://journals.plos.org/plosone/s/figures#loc-tools-for-figure-preparation.

NAAS will assess whether your figures meet our technical requirements by comparing each figure against our figure specifications.

# Author comment: Author response to Decision Letter 1

**DOI:** 10.1371/journal.pone.0351637.r002

13 May 2026

Dear Academic Editor and Reviewers,

We sincerely thank the Academic Editor and the Reviewers for their careful evaluation of our manuscript and for their constructive comments.

We have carefully revised the manuscript in response to these comments. A point-by-point response to all comments has been prepared and uploaded as a separate “Response to Reviewers” file. The revised manuscript has also been updated accordingly, and the main changes have been highlighted in yellow for ease of review.

In response to the reviewers’ comments, we reran the model-selection analysis after adding mean arterial pressure (MAP) and correcting the coefficient extraction procedure. Consequently, the selected ModelBasic_CST was updated to include sex, A2 deformation amplitude, and HC deformation amplitude. The Abstract, Results, and Discussion sections have been revised accordingly.

In accordance with the comments from PLOS ONE, we removed the Age column from the publicly shared dataset to protect participant privacy.

We also corrected errors in the signs of the coefficients for IOP and A1 length in Table 3. Specifically, in the single-parameter LMMs, the coefficients for both IOP and A1 length were positive, not negative. The corresponding descriptions in the Results and Discussion sections have been revised accordingly to ensure consistency with the corrected Table 3 and to avoid misinterpretation.

We hope that the revised manuscript is now suitable for publication in PLOS ONE.

Sincerely,

Yuta Nakaniida

Attachment Submitted filename: 02_Response to Reviewers.docx

# Peer review: Decision Letter 1

**DOI:** 10.1371/journal.pone.0351637.r003

**Authors:** Daisuke Nagasato

31 May 2026

Relationship between Corvis ST tonometry parameters and mean blur rate in the optic nerve head tissue area in healthy eyes

PONE-D-26-08321R1

Dear Dr. Nakaniida,

We’re pleased to inform you that your manuscript has been judged scientifically suitable for publication and will be formally accepted for publication once it meets all outstanding technical requirements.

Within one week, you’ll receive an e-mail detailing the required amendments. When these have been addressed, you’ll receive a formal acceptance letter and your manuscript will be scheduled for publication.

An invoice will be generated when your article is formally accepted. Please note, if your institution has a publishing partnership with PLOS and your article meets the relevant criteria, all or part of your publication costs will be covered. Please make sure your user information is up-to-date by logging into Editorial Manager at Editorial Manager® and clicking the ‘Update My Information' link at the top of the page. For questions related to billing, please contact billing support.

If your institution or institutions have a press office, please notify them about your upcoming paper to help maximize its impact. If they’ll be preparing press materials, please inform our press team as soon as possible -- no later than 48 hours after receiving the formal acceptance. Your manuscript will remain under strict press embargo until 2 pm Eastern Time on the date of publication. For more information, please contact onepress@plos.org.

Kind regards,

Daisuke Nagasato

Academic Editor

PLOS One

Additional Editor Comments (optional):

Reviewers' comments:

Reviewer's Responses to Questions

**Comments to the Author**

1. If the authors have adequately addressed your comments raised in a previous round of review and you feel that this manuscript is now acceptable for publication, you may indicate that here to bypass the “Comments to the Author” section, enter your conflict of interest statement in the “Confidential to Editor” section, and submit your "Accept" recommendation.

Reviewer #1: All comments have been addressed

Reviewer #2: All comments have been addressed

**********

2. Is the manuscript technically sound, and do the data support the conclusions?

The manuscript must describe a technically sound piece of scientific research with data that supports the conclusions. Experiments must have been conducted rigorously, with appropriate controls, replication, and sample sizes. The conclusions must be drawn appropriately based on the data presented.

Reviewer #1: Yes

Reviewer #2: Yes

**********

3. Has the statistical analysis been performed appropriately and rigorously?

Reviewer #1: Yes

Reviewer #2: Yes

**********

4. Have the authors made all data underlying the findings in their manuscript fully available?

The PLOS Data policy requires authors to make all data underlying the findings described in their manuscript fully available without restriction, with rare exception (please refer to the Data Availability Statement in the manuscript PDF file). The data should be provided as part of the manuscript or its supporting information, or deposited to a public repository. For example, in addition to summary statistics, the data points behind means, medians and variance measures should be available. If there are restrictions on publicly sharing data—e.g. participant privacy or use of data from a third party—those must be specified.

Reviewer #1: Yes

Reviewer #2: Yes

**********

5. Is the manuscript presented in an intelligible fashion and written in standard English?

PLOS ONE does not copyedit accepted manuscripts, so the language in submitted articles must be clear, correct, and unambiguous. Any typographical or grammatical errors should be corrected at revision, so please note any specific errors here.

Reviewer #1: Yes

Reviewer #2: Yes

**********

6. Review Comments to the Author

Please use the space provided to explain your answers to the questions above. You may also include additional comments for the author, including concerns about dual publication, research ethics, or publication ethics. (Please upload your review as an attachment if it exceeds 20,000 characters)

Reviewer #1: After carefully reviewing the revised manuscript and the authors’ detailed responses to the reviewers’ comments, I believe that the manuscript has been substantially improved and now meets the standard for publication. Although some limitations remain, these have been appropriately acknowledged by the authors and do not significantly undermine the overall value of the study.

Therefore, I consider that the manuscript is acceptable for publication in its current form.

Reviewer #2: I appreciate the authors' responses, which have addressed all of my concerns. The manuscript has been sufficiently improved.

**********

7. PLOS authors have the option to publish the peer review history of their article (what does this mean?). If published, this will include your full peer review and any attached files.

If you choose “no”, your identity will remain anonymous but your review may still be made public.

**Do you want your identity to be public for this peer review?**  For information about this choice, including consent withdrawal, please see our Privacy Policy.

Reviewer #1: No

Reviewer #2: No

**********

# Editor report: Acceptance letter

**DOI:** 10.1371/journal.pone.0351637.r004

**Authors:** Daisuke Nagasato

PONE-D-26-08321R1

PLOS One

Dear Dr. Nakaniida,

I'm pleased to inform you that your manuscript has been deemed suitable for publication in PLOS One. Congratulations! Your manuscript is now being handed over to our production team.

At this stage, our production department will prepare your paper for publication. This includes ensuring the following:

* All references, tables, and figures are properly cited

* All relevant supporting information is included in the manuscript submission,

* There are no issues that prevent the paper from being properly typeset

You will receive further instructions from the production team, including instructions on how to review your proof when it is ready. Please keep in mind that we are working through a large volume of accepted articles, so please give us a few days to review your paper and let you know the next and final steps.

Lastly, if your institution or institutions have a press office, please let them know about your upcoming paper now to help maximize its impact. If they'll be preparing press materials, please inform our press team within the next 48 hours. Your manuscript will remain under strict press embargo until 2 pm Eastern Time on the date of publication. For more information, please contact onepress@plos.org.

You will receive an invoice from PLOS for your publication fee after your manuscript has reached the completed accept phase. If you receive an email requesting payment before acceptance or for any other service, this may be a phishing scheme. Learn how to identify phishing emails and protect your accounts at https://explore.plos.org/phishing.

If we can help with anything else, please email us at customercare@plos.org.

Thank you for submitting your work to PLOS ONE and supporting open access.

Kind regards,

PLOS ONE Editorial Office Staff

on behalf of

Dr. Daisuke Nagasato

Academic Editor

PLOS One
